# Hookah (Shisha, Narghile) Smoking and Environmental Tobacco Smoke (ETS). A Critical Review of the Relevant Literature and the Public Health Consequences

**DOI:** 10.3390/ijerph6020798

**Published:** 2009-02-23

**Authors:** Kamal Chaouachi

**Keywords:** Hookah, shisha, narghile, tobacco, smoking, environmental tobacco smoke (ETS), particles, public health

## Abstract

Hookah (narghile, shisha, “water-pipe”) smoking is now seen by public health officials as a global tobacco epidemic. Cigarette Environmental Tobacco Smoke (ETS) is classically understood as a combination of Side-Stream Smoke (SSS) and Exhaled Main-Stream Smoke (EMSS), both diluted and aged. Some of the corresponding cigarette studies have served as the scientific basis for stringent legislation on indoor smoking across the world. Interestingly, one of the distinctive traits of the hookah device is that it generates almost no SSS. Indeed, its ETS is made up almost exclusively by the smoke exhaled by the smoker (EMSS), i.e. which has been filtered by the hookah at the level of the bowl, inside the water, along the hose and then by the smoker’s respiratory tract itself. The present paper reviews the sparse and scattered scientific evidence available about hookah EMSS and the corresponding inferences that can be drawn from the composition of cigarette EMSS. The reviewed literature shows that most of hookah ETS is made up of EMSS and that the latter qualitatively differs from MSS. Keeping in mind that the first victim of passive smoking is the active smoker her/himself, the toxicity of hookah ETS for non-smokers should not be overestimated and hyped in an unscientific way.

## 1. Introduction

### 

#### Hookah Smoking as a New Public Health and Environmental Research Field

**Figure 1.**
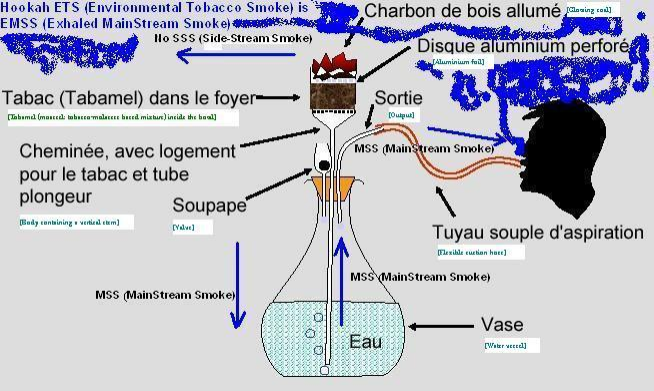
The hookah (narghile, shisha) operating procedure and the diverse smoke flows (Chaouachi, Cours du DIU de Tabacologie, Universite Paris XI). **English terms (from left to right):** TOBAMEL (moassel: tobacco-molasses based mixture) inside the bowl [for “Tabac (tabamel)...”]; BODY CONTAINING A VERTICAL STEM [for “Cheminée avec logement...”]; VALVE [for “Soupape”]; WATER VESSEL [for “Vase” and “Eau”]; FLEXIBLE SUCTION HOSE [for “Tuyau d’aspiration souple”]; OUPUT [for “Sortie”]; ALUMINIUM FOIL (punched with holes) [for “Disque d’aluminium perforé”]; GLOWING COAL [for “Charbon de bois allumé”].

Hookah (narghile, shisha) smoking is an ancient mode of tobacco use which has not posed any particular public health problem over the past centuries [1] (Figure 1).

Interestingly, its recent Middle East revival – and its transformation into a worldwide fashionable habit – coincided with the emergence of the ETS (Environmental Tobacco Smoke) – taken as synonymous to the SHS (Second Hand Smoke)-question in the early 1980s, particularly in North America and Western Europe. The reasons behind the growing popularity in the latter regions were early described elsewhere [2]. Fifteen motives, at least, could be identified. One of them assumes that the powerful anti-cigarette campaigns of the last decades would have, as a backlash effect, pushed a certain number of cigarette smokers towards a tobacco use mode viewed as less hazardous to health and, above all, less addictive. A recent study confirms the latter aspect, as it was found that more than 90% of so-called “mild smokers” (3 pipes or less per week) and about 50% of the so-called “moderate” ones (3 to 6 pipes per week) are considered as non dependent [3]. Existing scenarios for such a complex health and socio-cultural phenomenon, never witnessed before in the archives of tobacco research, have been proposed and revisited [4]. In these conditions, critical and comprehensive reviews of what sound scientific research says about hookah smoking health effects were necessary. The first one ever carried out can be found in a tobaccology (tobacco science) thesis dated 1998 and reworked later into a doctoral thesis [5]. This first review was updated several times and took the form, in particular, of a tetralogy on hookah and health [6]. Most recently, two teams from Asia and Africa have elicited a substantial advancement of research in this field. The first one analysed the potential health hazards associated with radioactivity in the smoking mixtures used in the narghile and found no great differences with cigarettes [7]. The other team led the first aetiological study on hookah smoking and cancer thanks to their fine selection of exclusive/ever hookah smokers who have been using, for decades, huge amounts of tobacco in their pipes. Using CEA as a cancer biomarker, they found a weaker association than that in cigarette smoking [8]. Such a study helped in clearing up a growing confusion caused, among others, by the dismissal of early biomedical and anthropological research on the subject [5].

For instance, a recent review in the *Journal of the International Union of Tuberculosis and Lung Disease* openly declares that hookah smoking might well be a ploy of the tobacco industry. Apart from various misquotations and errors, it also presents a selection of cancer studies with no comment on the fact that the participants were simultaneous or former smokers of cigarettes or other products [9]. As for the purported link to the Tobacco Industry, a physician native of Nepal wrote, as early as 1962, to the *British Medical Journal,* regretting and lamenting the arrival of cigarettes in remote regions where only hookah had been traditionally used before. He said, after noting that “this form of smoking is less harmful than smoking cheap-brand cigarettes, as done by the majority of people in [his] country”: “I wonder how these representatives of the tobacco industry manage to reach the almost inaccessible hilly regions in the country”[10].

Another difficulty in this novel field of research that has received the attention and the funding of world organisations, has been frequent publications bias, not to mention linguistic bias or what some research labels “institutional provincialism” and other forms of ostracism [11,12]. Since a living hookah is not a mere “water pipe” laid on the table of a chemistry laboratory but generally involves a complex human and social situation, a short overview of recent findings about the health effects of hookah active smoking, i.e. exposure of the smoker to MSS, is necessary. Indeed, the first victim of “passive smoking” is the active smoker herself/himself [13].

Recently published studies from Asia and Africa are shedding new light on the potential diseases associated with hookah use through exposure to its MSS (active smoking). On the whole, exhaled CO by active smokers may be high in certain circumstances [1,14] and lung problems may arise in the case of heavy use. Metabolic effects could be similar to those observed in cigarette smokers [15,16]. As a general rule, studies on respiratory effects have been contradictory in the past. For instance, in Tunisia, Ourari *et al.* have compared the cytology of the BAL (bronchoalveolar lavage) fluid (macrophages, lymphocytes, neutrophils et eosinophils) and the lung function in 30 narghile users and 10 cigarette smokers. They found that regular use of narghile induces a rise in the overall cell number at the level of the BAL. However, it does not seem to bring about significant changes in lung function parameters when compared to cigarettes. The FEV1 (Forced Expired Volume in 1 second) and lung capacity were significantly higher [17]. However, a Syrian team has been able to shed new light on the respiratory effects of heavy narghile use among daily female users of tumbak (pure moistened tobacco, no molasses, no flavours, no glycerol). This was made possible thanks to their clear selection of exclusive/ever users. The researchers reported a higher proportion of chronic bronchitis in narghile smokers and quasi-permanent alteration of maximum Maximal Mid-Expiratory Flow (MMEF 25%–75%) in narghile smokers when compared with cigarette smokers. Nonetheless, FEV1 was more altered in cigarette smokers [18]. The risk of tuberculosis was highlighted and the lesser carcinogenic effect of narghile smoking brought out again [18,19]. This is in agreement, to a certain extent, with the recent aetiological study carried on in Pakistan [8]. As for communicable diseases, there has been some confusion now addressed in a recent review [20].

The hookah practice is striking by its great social, cultural, linguistic, material and geometrical diversity. For instance, the device bears such names as narghile (spelled “nargile” in Turkish), shisha, hookah, goza, madâ’a, qalyân, *etc.* Most of these terms refer to the water vessel in the corresponding languages. Three main smoking mixtures have been clearly identified: moassel, tumbak and jurak [5]. One of the most important consequences of such a diversity is that the chemistry of smoke will be extremely different according to pipes, products and context. Reducing such a complexity through the use of an arbitrary name like “waterpipe” actually qualified for a scientific nominalism (see Glossary).

Furthermore, a widely endorsed functionalist bias occurred when complex social situations (those in which a hookah session takes place) were reduced to a laboratory model based on a “waterpipe” smoking machine supposed to replicate the emissions of toxicants actually inhaled by smokers during such social events. These methods have been criticised and, as an example, it was recalled that the FTC (Federal Trade Commission) and ISO norms suggest the use of a 1 minute machine smoking interval between 2 puffs in the case of cigarettes for which the duration of a laboratory session barely exceeds 5 minutes. However, and by a striking contrast, the “waterpipe” used in the laboratory was based on 171 steady puffs drawn every 17 seconds, i.e for one full hour, with the charcoal (heating source) on the same point over the smoking mixture. In these conditions, the nature and advertised yields of the measured toxicants in the smoke are highly questionable [4,21].

Another frequent confusion relates to the smoked products. As said before, these are of three main types – tumbak, moassel and jurâk – and they produce different chemical reactions in each case. However, the authors of recent studies in key journals *(Nicotine and Tobacco Research; American Journal of Health Behaviour)* mistook one product for the other: tumbak for moassel or jurak. Also, by taking the above-mentioned smoking machine as a good approximation of the reality of hookah smoking, and ignoring the qualitative differences between hookah smoke and cigarette smoke, they went so far as to consider that, in “a standard waterpipe session”, a series of toxicant yields (nicotine, heavy metals, “tar”) are doses actually inhaled by smokers in the real world [22,23].

In fact, the smoke of hookah is chemically much less complex than that of cigarettes. This is due to the much lower temperatures to which the tobacco-molasses mixture is subjected: actually hundreds of degrees below that of cigarettes. Notably, and in striking contrast with ordinary cigarettes, a great part of the smoke is made up of water and glycerol when moassel is used [8]. It had been previously found that the water-soluble portion of cigarette smoke represented 38% of the particulate matter [24]. Interestingly, Middle East researchers have subsequently estimated the overall shisha water-filtration rate to be 38%, and concluded that shisha smoke, with only 142 compounds detected in a pipe filled with jurak (a mixture of 15% of tobacco leaves and 47% carbohydrates (glucose)), is actually far less complex than cigarette smoke [25]. This figure can be compared with the 4,700 substances identified so far in cigarette smoke [26].

**Figure 2.**
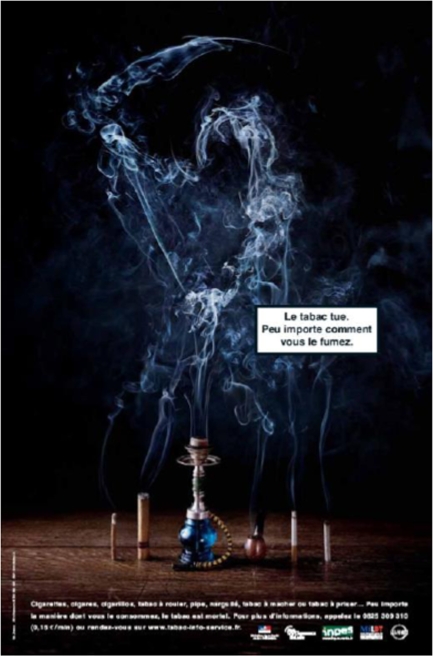
Poster of the French INPES (Institut National pour la Prevention et l’Education a la Sante). This visual aid was used during the 2005 “World No Tobacco Day” campaign sponsored by the WHO. It shows a huge cloud of dense smoke (supposedly ETS) stemming from a hookah and featuring the spectrum of death.

For almost one decade now, public health organisations have failed to properly address the ever-growing world hookah epidemic despite their focus on hookah ETS hazards. A first example is a campaign poster designed by the French INPES (Institut National pour la Prevention et l’Education a la Santé) which was used during the 2005 “World No Tobacco Day” campaign sponsored by the WHO. The poster shows an important cloud of thick smoke stemming from a hookah and featuring the spectrum of death (Figure 2).

**Figure 3.**
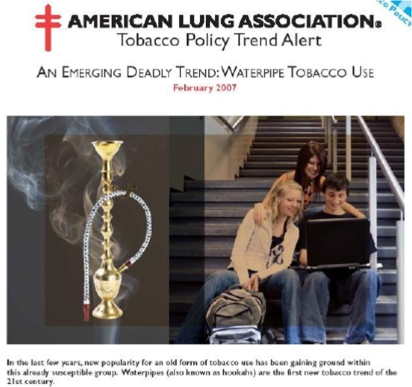
Cover of the American Lung Association’s report on “waterpipe”. It shows a small-size hookah generating SSS on its own (American Lung Association. An Emerging Deadly Trend: Waterpipe Tobacco Use. Feb 2007).

**Figure 4.**
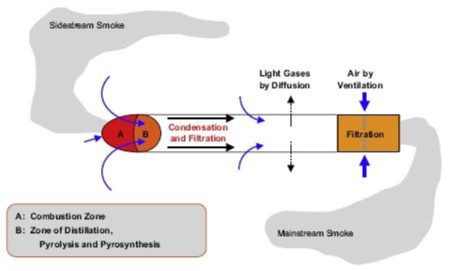
Main-Stream Smoke and Side-Stream Smoke in a burning cigarette (Thielen *et al.* [26]).

Another example is a report by the American Lung Association whose cover shows, once again, a small-size hookah generating SSS on its own (Figure 3). Unfortunately, what the tobacco experts who prepared both reports ignored is that, in contrast with cigarettes (Figures 1 and 4), a hookah does not generate such a side-stream smoke.

Yet, researchers had pointed out that “one of the only articulated benefits to this tobacco alternative is the minimal release of side-stream smoke, which would ultimately place by-standers at risk for ETS exposure” [27].

#### Overview of Landmark Studies on Cigarette ETS

Cigarette ETS is classically defined as a combination of SSS emitted from the burning end of a cigarette and the remainder of EMSS, i.e. the MSS exhaled by the smoker (Figure 3). It was also proposed that pregnant women smoking be considered as ETS to which the foetus is exposed [28]. SSS constitutes about 85% of the smoke present in a room where active smokers smoke, and contains many potentially toxic components [29]. Many authors insist on the issue of dilution and aging (from minutes to hours) of the resulting smoke in the environment [26,30]. Indeed, the corresponding chemical process imply that the composition of the two phases of the smoke (gaseous/vapour and particulate) will undergo important changes (nicotine, radicals, *etc.*) and the overall cytotoxicity will decrease [26]. ETS is made up of millions of particles of different sizes in which, among others, nicotine, hydrocarbons, phenols, heavy metals and glycerol can be found.

Prior to presenting what biomedical research says about the consequences of hookah ETS, it appears necessary to offer an overview of what is known about cigarette ETS. Some authors regret that a part of the literature on (cigarette) ETS is based on passionate assertions [31]. However, for the WHO, “rigorous research leaves no doubt” that SHS is injurious to health. It would cause cancer, as well as many serious respiratory and cardiovascular diseases in children and adults, often leading to death. There would be no safe level of human exposure to tobacco SHS. According to the same organisation, these “indisputable conclusions” would be “backed up by “extensive rigorously reviewed and published research results, over many years” and three major publications are relevant: a monograph of the IARC (International Agency for Research on Cancer); a report from the California Environmental Protection Agency and another one from the US Surgeon General [32–35]. Indeed, for almost three decades, almost 100 epidemiological studies have analysed the risk of lung cancer and coronary heart diseases for non-smokers exposed to ETS [36]. A European report also mentions earlier documents published in the 1980s and concludes that the series of epidemiological studies following them provides “compelling evidence of a causal relationship” between ETS and respiratory outcomes, and cardiovascular effects and lung cancer [37].

By 1993, Huber *et al.,* among others, have severely criticised the flaws contained in the EPA report published one year earlier, which claimed that SHS causes 3,000 deaths a year and classified it as a class A carcinogen. The critics found that this report “ignored classic criteria for cause-and-effect relationships employed by the scientific community” and that “concentrations of constituents also vary widely from time to time and from place to place. Furthermore, compared to other kinds of tobacco smoke, only a small fraction of the constituents of MSS and of SSS potentially present in ETS have ever been quantifiably identified in the real-world air to which the non-smoker is exposed” [38,39]. Others have pointed out that “consistent epidemiologic data indicate that active smoking of some 4–5 cigarettes per day may not be associated with a significantly increased risk of lung cancer” and since “average doses of ETS to nonsmoking subjects in epidemiologic studies are several thousand times less than this reported intake level, the marginal relative risks of lung cancer and other diseases attributed to ETS in some epidemiologic studies are likely to be statistical artifacts, derived from unaccounted confounders and unavoidable bias” [30].

A European multi-centre case control study involving 650 patients with lung cancer and 1,542 controls, found no association between childhood exposure to ETS and risk for lung cancer [40]. However, a large European prospective study a few years later concluded that ETS is a risk factor for lung cancer and other respiratory diseases, particularly in ex-smokers [41]. Adlkofer, a researcher who has studied biological effects of ETS for 20 years, has led a review on the associations between ETS and lung cancer. He concluded that “the average intake of toxic and genotoxic compounds due to ETS exposure is that low that it is difficult, if not impossible, to explain the increased risk of lung cancer as found in epidemiological studies” [31].

Against the backdrop of the WHO Tobacco Free World agenda, bans on indoor (including private places) and even outdoor smoking (i.e. in the open air) are multiplying, endorsing compliance with recommendations being included in supranational legislation such as the FCTC (Framework Convention for Tobacco Control). It has now become commonplace to see crowds of smokers “occupying” terraces and patios of cafes and restaurants in countries where bans on indoor smoking have been enforced in recent years. As a consequence of this spatial shift, some studies also focus on OTS (Outdoors Tobacco Smoke). For example, in one of them, “some average concentrations over the duration of a cigarette and within 0.5 m exceeded 200μg/m^3^”. However, it was noted that “OTS levels in a constant upwind direction from an active cigarette source were nearly zero and that OTS levels also approached zero at distances greater than approximately 2 m from a single cigarette”. This study concludes that OTS may therefore be a nuisance or a hazard in certain conditions [42]. Some anti-smoking organisations have perhaps gone too far in trying to change lifestyles. Social scientists have recently tried to understand why smoking, which has been part of everyday life during the 20^th^ century, including at work, has suddenly become unacceptable and sometimes banned. Smoking, they insist, “should be understood as a practice with diverse cultural meanings, and its regulation located within the context of a longstanding and dynamic moral discourse, of which scientific and medical discourse is only one aspect” [43].

Some scientists have also tried to demonstrate that bans have a direct positive impact on populations’ health. In a study referred to as the “Helena study”, the authors aimed “to determine whether there was a change in hospital admissions for acute myocardial infarction while a local law banning smoking in public and in workplaces was in effect”. They concluded that “laws to enforce smoke-free workplaces and public places may be associated with an effect on morbidity from heart disease” [44]. This study has received a great deal of publicity. However, it was also criticised. In particular, one researcher noted that “the drop in heart attacks is based on very few cases” and that “the reported difference could easily be due to chance or to some uncontrolled factor” [45]. In 2008, and in a similar situation to that analysed in the Helena study, the Scottish government declared that the smoking ban enforced one year before in that country had also led to a dramatic fall in hospital admissions for acute coronary syndrome. However, “the latest figures suggest a rise of 7.8 per cent in the second year of the ban, cancelling out the earlier drop [...] This seems to be backed up by hospital data from England and Wales, which have failed to show a significant reduction in incidence of acute coronary syndrome since these two countries followed Scotland and went ‘smoke free’ in 2007 [46].

Concerning the potential associations between cardiovascular diseases, a critical review was published in 1995. It concluded that epidemiological reports are inconclusive and that “such equivocations likely result from the presence of contrasting protective or aggravating confounders, of which more than 200 have been reported in the literature – confounders that were not and could not be adequately controlled by any epidemiologic study. By scientific standards, the weight of evidence continues to falsify the hypothesis that ETS exposure might be a CHD risk factor [47]. More recently, another critical analysis lamented inaccurate claims by anti-smoking organisations that a single, acute, transient exposure to SHS can cause severe and even fatal cardiovascular events in healthy non-smokers [48]. Also, a comprehensive review of the epidemiologic evidence relating stroke to exposure to ETS in lifelong non-smokers concluded that so far the association is only suggestive of a possible causal relationship [49].

In a study based on a long follow up of a wide cohort of Californians, two researchers found no causal relation between ETS and tobacco related mortality although they did not rule out a small effect. They considered that the association between exposure to ETS and coronary heart disease and lung cancer is “considerably weaker than generally believed” [50]. These unexpected results have been hardly accepted by anti-smoking organisations; to the point that the first author had to publish a defence of that work in which he addresses, among others, “the omission of [his] research from the 2006 Surgeon General’s Report on Involuntary Smoking and the inclusion of it in a massive U.S. Department of Justice racketeering lawsuit”[51]. Also, the unusual flow of electronic online “rapid responses” to the California study were scrutinised by two social scientists who concluded that “the public consensus about the negative effects of passive smoke is so strong that it has become part of a regime of truth that cannot be intelligibly questioned”[52].

In Europe, the situation reached the point where a scholar and top tobacco authority in his country reacted to official statistics reporting a large increase of ETS-induced death toll in Europe [53]. He found that among the 5,863 estimated deaths in the report entitled “Lifting the Smokescreen”, 4,749 concerned everyday smokers. Furthermore, he pointed out that the 1,114 “non-smokers” included all former smokers as well. The remaining risk of the latter, he added, could not be ascribed to ETS. It is also noteworthy that the conclusions of this European report have been decisive to passing laws banning smoking in public places [13].

#### Recent Concern about Hookah ETS and Differences with Cigarette ETS

In this context of a serious scientific debate over the actual effects of cigarette ETS on one hand, and, on the other, the social consequences of smoking bans (from cafes to homes), the present review on hookah smoking and ETS has proved to be more necessary than ever. First, hookahs are known to emit clouds of smoke. Second, there is also a not less hot controversy over the effects of its MSS, i.e. the one related to active smoking. Third, hookah smoking is enjoying a growing popularity across the world. For some, it represents the revival of an old social and cultural tradition, while others view it as the discovery of another way of smoking. For anti-smoking organisations, it is a counterintuitive epidemic, seen as the first tobacco epidemic of the 21^st^ century. Their researchers first thought that the cigarette ETS paradigm was valid for any kind of smoking so they applied it to hookah smoking. However, they realised that while all the corresponding theory was mainly focussing on (cigarette) SSS, the given models were not going to be of a great help because hookah is known for not generating such “lateral” smoke (Figures 1, 4). Only one team of researchers clearly pointed out the consequences of this fact [27].

Interestingly, in one of its reports, the WHO offers a universal definition of ETS/SHS: *“Second-hand tobacco smoke (SHS) refers to the smoke from burning tobacco products, generated by people smoking them”* [54]. This is certainly true for cigarettes or cigars. However, the WHO experts have not realised that the smoking product (moassel) in the hookah bowl is not burned but only heated (below 200°C) to a great extent. This has been highlighted elsewhere [4]. A practical consequence of the emergence of hookah smoking as a public health problem is that WHO’s definition of ETS needs to be changed. Yet, an Indian team had relevantly pointed out that bidis and hookah SSS would differ from standard cigarettes “due to differences in tobacco processing, burning rate and temperature, and the use of additives for burning tobacco”[29].

Most of the few publications directly connected with hookah ETS have so far focussed on the analysis of particles of different sizes present in EMSS. These studies are reviewed in the present work. Confusion has reached a substantial level since the WHO experts declared that: “Second-hand smoke from waterpipes […] poses a serious risk for non-smokers” [4,55]. Such a statement refers to a publication of the US-Syrian Centre for Tobacco Studies, chief co-author of the WHO report [56]. Another WHO report, prepared by a US-Egyptian team, while acknowledging that shisha ETS has been addressed in a very limited number of studies, states: “Yet, there is strong evidence that exposure to waterpipe smoking is as harmful as the exposure to cigarette smoking, if not more harmful” [28]. Most recently, the *International Journal of Tuberculosis and Lung Disease* has published a review in which, among noticeable errors, the same claim is made [9].

#### Difference between ETS and MSS

Cigarette ETS and MSS are different, as many authors have pointed out [30,31,57,58]. The WHO stresses that “SHS contains thousands of identified chemicals, at least 250 of which are known to be carcinogenic or otherwise toxic. Among those chemicals and toxins are the deadly, odourless, colourless gas carbon monoxide (CO), increased levels of acetaldehyde, acrolein, formaldehyde and many other substances. When inhaled, these poisons are concentrated and quickly spread throughout the body, leading to a range of serious diseases” [35]. Warnings were issued early on that “risk extrapolation from active smoking to passive smoking is of doubtful value” [57]. Concerning hookah ETS, it appears that it considerably differs from cigarette ETS for three main reasons. The first reason is that a hookah does not generate SSS. The second reason is that, when comparing both systems, the temperatures at stake are very low in hookah smoking. Indeed, they barely exceed 200°C (in the case of the widely used moassel/tobamel; different from other products) whereby chemical reactions will differ completely. The result is distillation (to a certain extent) instead of pyrolysis as it occurs in cigarettes, where the tip of the latter reaches 900°C. The third reason is the ageing of smoke. For instance, and taking the example of cigarette smoke, the “decrease of NO in the fresh smoke is accompanied by an increase of NO_2_. NO_2_ concentration reaches a maximum after about 1 min and then it decreases. CH_3_OH concentration in the smoke is stable for about 10 s, after which it decreases parallel to the NO_2_ concentration. That means there will be a reaction between the nitrogen oxides and CH_3_OH, resulting in artificial formation of methyl-nitrite CH_3_NO_2_, a component which is not present in fresh tobacco smoke” [59].

#### Ageing

Indeed, while ageing, smoke undergoes numerous transformations which adds to its complexity. However, researchers noted that one tobacco company (Philip Morris) carried out a series of studies on the toxicity of freshly generated SSS. When considering total particulate matter, the results show that this SSS would be up to four times more toxic than MSS. They regret that none of these were ever published [60]. In this case, the debate is about the SSS toxicity of a smoking instrument (the cigarette) whose length is about 10 cm. So, the issue of smoke ageing which is central in the assessment of ETS toxicity, should be considered with even more caution in the case of hookah smoking simply because, and as strange as it may be, the smoke covers a distance of about 25 times that of cigarette. It is clear that from the production site (the bowl) to the mouth of the smoker, the smoke has to go down the long vertical stem (sort of inverted chimney), bubble through the water and build up above the surface of the latter (as in an air lock or double door system) between two puffs. During each puff, the smoke is then introduced into the long suction hose. An interesting relevant phenomenon was discovered two decades ago by a researcher: the accumulation of particles in the void volume of the 10 cm cigarette during the 1-min smoulder period between puffs [61,62]. Therefore, most of the time, the smoker inhales (when it does), and apart from the first puff (which will be diluted with more air than the following ones), the smoke that reaches the hookah user’s mouth is far from being fresh. It is aged, considerably aged, smoke.

In view of all the above described distinguishing traits, a central objective of the present review is to determine whether hookah ETS represent a “serious risk” to non-smokers. It is recalled, once again, that what is at stake here is “passive smoking”, not active smoking. The latter has also been surrounded by a wide confusion. Consequently, the focus of the present review is on EMSS.

## 2. Results and Discussion

Beside the dearth of publications on hookah ETS, one major difficulty has been that some of the studies deemed relevant for this review, do not clearly differentiate active from passive smoking. Most of the selected documents are of an epidemiological or experimental nature. Yet, a fair number of them are recent, a fact reflecting the public health official’s concern over, if not hookah ETS, compliance with the WHO Tobacco Free world agenda and the corresponding FCTC (Framework Convention for Tobacco Control) [35]. Most of the identified studies come from Asia (India, Arabia, Syria, Lebanon), Africa (Egypt) and, recently, from the USA or US-funded institutions. Only two were led in Europe (Switzerland, France). In India, a team has usefully reviewed the literature related to the health (respiratory) effects of ETS [29]. In Lebanon, two epidemiological surveys were identified [63,64]. It is also noteworthy that for most of the experimental studies, the focus has been on particles (PM_2.5_, PM_10_ and Ultra-Fine Particles, i.e. sub-micrometer sized particles); CO (probably because of the charcoal heating source which clearly distinguishes it from cigarettes). For other chemicals (PAH, aldehydes, *etc*.), only inferences can be made from pioneering studies carried out these last years on cigarette MSS vs. EMSS [58,65–68].

### 

#### Overview of ETS Markers

When assessing the effect of ETS in general, researchers work either on environmental markers (particularly: CO, nicotine in the air, volatile organic compounds, particulate matter, *etc.*) or they may wish to quantitate biomarkers. Vapour phase and particle phases markers are distinguished. Markers of the former are nicotine, carbon monoxide (CO), 3-ethenylpyridine, nitrogen oxides, pyridine, aldehydes, acrolein, benzene, toluene, *etc.* For the particle phase, common markers are: RSP (Respirable suspended Particles), solanesol, *N*-nitrosamines, cotinine, chromium, potassium. However, the most commonly used are RSP, CO and nicotine [69]. The validation of a biomarker is a complex process, involving such criteria as specificity (to tobacco smoke), sensitivity, dose-response relationships, inter/intra-individual variability, kinetics, confounders, *etc.* [36]. CO absorption certainly reflects acute exposure to ETS. However nicotine and its metabolite cotinine are the best markers currently available [70]. Indeed, cotinine (blood, saliva, or urine) is seen as the apparently most specific and sensitive ETS biomarker [71].

### 2.1. Exhaled MainStream Smoke (EMSS) Oriented Studies (Cigarette and Hookah)

Only MSS and SSS have been so far clearly defined and it was stressed that they differ chemically from each other. It appears that EMSS, whenever inhaled by non-smokers, will interact with internal tissues and enzymes. Indeed, MSS is stripped, within the smoker’s respiratory tract of many of its volatile chemical compounds. What remains as EMSS is only “small amounts of residual altered mainstream smoke particulates, saturated with water vapor by their passage through the respiratory system and dramatically reduced in volatile chemical constituents, as well as some gas phase residual constituents” [38].

Borgerding has summarised the findings of previous research of the 1990s by stating that cigarette EMSS, i.e. exhaled, “respiratory tract filtered” mainstream smoke, contributes between 15% and 43% of the particulate matter of ETS and between 1% and 13% of the vapour phase, the remaining originating from SSS [59]. Several studies, including reviews, on cigarette EMSS were identified. They are even more interesting because, unlike results focussing exclusively cigarette SSS, cigarette MSS/EMSS ratios and the related phenomena (e.g. particle growth because of the humid environment of the respiratory tract or the presence of glycerol) are –this is assumed- relevant to hookah EMSS. For instance, recent trailblazing studies aimed to determine levels of polycyclic aromatic hydrocarbons, carbonyl compounds, benzene, toluene and hydroxybenzenes in cigarette EMSS [58,65–67]. Its chief author (Moldoveanu) has also experimentally clarified the differences between MSS and EMSS. The retention rate of 160 compounds from MSS by eight human subjects was found to differ from one compound to the other (ranges: 5–10% to 90–100%). The less retained compounds (below 33%) were mainly long-chain hydrocarbons (saturated or squalene type) and phytosterols [68]. It should be recalled that in the past, studies on deposition of tobacco smoke in smokers’ respiratory tract have generally involved methods that interfere with normal smoking [72].

Baker and Dixon have carried out an important review of the literature covering more than one century. They found that an average of 60 to 80% of cigarette MSS particulate matter is retained in the lungs after inhalation. For nicotine, carbon monoxide, nitric oxide, and aldehydes, the total retentions are of the order of 90–100, 55–65, 100, and approximately 90%, respectively. As for the retention rate in passive smokers, they are smaller: 71–81% for nicotine, and 11–59% for particulate matter retention [73]. Two years earlier, Bernstein had published an important review, however of a smaller size and focussing on the influence of particle size, puff volume, and inhalation pattern on deposition inside the respiratory tract. He found, among other things, that the cigarette smoke particle size is in the same range as the minimum deposition particle size in the lung [61]. Baker and Dixon had taken note of that review and noted that “Bernstein concluded that particulate deposition within the lung would not change significantly when comparing low-yield/filterventilated cigarettes to higher yield/non-ventilated cigarettes, even if smoker compensation occurred”. However, they point out that the studies they have analysed in their own review suggest that “the presence of filter ventilation in the cigarette does decrease the retention of smoke particulate matter in the lung, which is at variance with Bernstein’s conclusion” [73]. In these conditions, references to Moldoveanu *et al.,* Baker and Dixon, and Bernstein, will be granted the deserved space in the results section. Finally, an important review on ETS was also identified. Edited by Rylander, its interest lies in the fact that it put “a special emphasis on the dose-response aspect and the relevance of the data for exposure to ETS under real life conditions” [74]. In fact, the “real life conditions” were, in many reported experiments, situations of extreme exposure, definitely not to be found tin everyday life. The cited document is a compiled and edited account of a high-level expert workshop on ETS, which included, among others, the researcher MAH Russell.

### 2.2. Epidemiological Studies Approaching Hookah ETS

Such studies aim at investigating the potential health hazards (otitis, asthma and other respiratory diseases), particularly threatening children exposed to tobacco smoke. Most of them come from Lebanon and India.

#### Middle East Studies

Surprisingly, and equating cigarette and hookah ETS by setting aside the discriminative SSS dimension, the US-Syrian centre states that: “the health effects of ETS exposure from water pipe on children have not yet been evaluated comprehensively, but they are likely to include many of those that result from exposure to cigarette smoke, including increased risk of ear and upper respiratory infection, asthma, and sudden infant death syndrome” [56]. Tamim *et al.* have authored two studies in this respect. In the first one, the health effects on Lebanese “students” (10–15 years old) were assessed “as to whether he or she suffered from respiratory tract ailments throughout the year (not seasonal), including nasal congestion or wheezing” [64]. In the other study, pre-school children’s exposure to cigarette and narghile ETS at home was evaluated [63]. This last study presents some problems because of a confusion between smoking products and because of its methodology. Notably, the questionnaire did not ask where the narghile was smoked. On the one hand, and in contrast with cigarette, a narghile is, once it is lit, generally not moved from one place to the other and from one room to the other in homes. On the other hand, it is generally smoked outdoors in Asia and Africa. This fact should have been taken into consideration.

In Egypt, the WHO EMRO report comments on three studies. The first one by El-Heneidy *et al.* (1999) showed that parental smoking is associated with early onset of asthma, severe atopic manifestations, higher levels of serum IgE, and reduced value of the predicted peak expiratory flow rate for age. Such an exposure would also be an important risk factor compared to other environmental pollutants. The second study is a Master thesis (Sherief, Al-Azhar University) showing that parental water pipe smoking was more prevalent among infants and children with chronic cough (than in the control group). The third study by Hessin *et al.* shows that ETS exposure significantly reduced the expected pulmonary function in healthy individuals [28]. In Saudi Arabia, researchers have found that there was a “high agreement” among interviewees that “smoke from cigarette (79.1%) and shisha (also called “kadu” there) (75.2%) cause eye irritation and cough” [75]. The US-Syrian Centre for Tobacco Studies has carried out a certain number of measurements. In one of them, out of 2,038 participants of a previous survey, 1,118 were non-smokers with a CO <= 10 ppm. Most participants were exposed to ETS and an association between the latter and impaired lung function in women was found [76]. The same centre conducted another survey pertaining to the same “Aleppo Household Survey” pool where a sample of 419 non-smokers was selected. It was found that the mean level of detectable cotinine in adult non-smokers was 1.7ng/ml (+/− 1.5) and that narghile smoking “does not seem to be an important source of ETS exposure” [77].

#### Indian Studies

A team found that exposure to ETS (nature of products and use modes unspecified) during childhood is an important risk factor for asthma and respiratory symptoms in non-smoking adults. After adjusting for age, gender, residence, atopy and cooking fuel used at home, it was deduced an OR of 1.378 (CI: 1.085–1.751) for household ETS exposure in childhood only and 1.165 (CI: 0.985–1.378) for household ETS exposure in adulthood only [78]. Other researchers in this country -after lamenting that lung cancer, which used to be rare in developing countries and is now fast emerging as a public health problem-, stressed that in Kashmir hookah is the most popular form of tobacco smoking. However, they note that it is “largely responsible for passive smoking of other family members, especially during winter months, when soot, smoke, and fumes from kitchens and various types of heating pollute the indoor air in ill ventilated and overcrowded dwellings” [79]. Another team described the “chillum” as being a pipe made of clay and in which “tobacco is burnt along with molasses and coal and smoked from the other end either directly at the mouth or through a long pipe with the smoke passing through a water container”. They found a strong association between cigarette ETS and lung cancer (OR: 5.1; CI: 1.5–17), while no association was seen for bidi or chillum” [80]. In a study not specific of hookah, another team concluded that “exposure to ETS during pregnancy is associated with higher risk of having a small-for-gestation baby” while interestingly noting about chillum and hookah that nicotine and other alkaloids in MSS or SSS from such smoking devices is “likely to be different than that known for standard cigarettes due to differences in tobacco processing, burning rate/temperature and design of the smoking product” [81].

In a multi-centre study, it was found that smoking both bidis and cigarettes, and exposure of non-smokers to ETS, were two important risk factors of COPD at all centres. The OR ranged from about 2 to 3.5 for different types of smoking. ETS exposure (although hookah was not discriminated from other smoking methods) was a stronger risk factor than solid fuel combustion and ETS exposure during adulthood was an important risk factor while exposure during childhood alone was not [82]. In another study involving a sample of 9,090 adolescent school children, ETS exposure (hookah not discriminated) was found to be associated with an increased risk of asthma (OR: 1.78; CI: 1.33–2.31). ETS increased morbidity and worsened control of asthma among adults. It was a significant trigger for acute exacerbation of asthma and increased bronchial hyper-responsiveness among healthy nonsmoking adult women. ETS led to subtle changes in airflow mechanics and exposure to it during childhood was strongly associated with an enhanced incidence of lung cancer (OR: 3.9; CI: 1.9–8.2). However, the observed risk was higher for ETS exposure through cigarettes as compared to bidis or chillum. For the authors, this difference was “consistent with the observation of comparative composition of MS and SS smoke from different tobacco products” [29].

### 2.3. Studies on Particles

#### Overview

Tobacco smoke is an aerosol that contains both gaseous and suspended particulate material. The particles are largely liquid droplets containing a wide variety of condensed organic compounds. Each compound in the smoke will partition between the gas and PM phases and will always seek a state of gas/particle equilibrium [83]. Particles contain tar, water, nicotine and other alkaloids [71]. PM_2.5_ and PM_10_ are particles with an aerodynamic diameter smaller than 2.5 μm, and 10 μm, respectively [69]. Mean particle size is 0.35–0.4 μm for MSS and 0.15–0.25 μm for SSS. Fresh SSS particles are smaller than MSS particles and SSS is generated at lower temperatures (Figure 4) [26]. Particulate matter would induce lung oxidative stress and impair balance between reactive oxygen species and reactive nitrogen species generation and oxidant defences” [84]. However, there are differences between the RSP (Respirable Suspended Particles) phases of ETS and MSS. This is because of different generation conditions and the fact that ETS is diluted and ages much more than MSS. “Even assuming similarities on an equal mass basis, ETS-RSP inhaled doses are estimated to be between 10,000- and 100,000-fold less than estimated average MSS-RSP doses for active smokers” [30].

Concerning mechanical aspects, Pankow has established that a compound such as nicotine can deposit in the respiratory tract by four different mechanisms:

*“1) Direct gas deposition (DGD) of the portion of the compound that is initially in the gas phase of the inhaled smoke; 2) evaporative gas deposition (EGD) of PM-phase compound by evaporation to the gas phase, then deposition; (3) particle deposition, evaporation from the deposited particle, then deposition from the gas phase (PDE); and (4) particle deposition with diffusion (PDD) into RT tissue. He adds that “Three of the mechanisms (DGD, EGD, and PDE) involve volatilisation from the PM phase. The relative importance of all the mechanisms is therefore greatly affected by the volatility of the compound from the PM phase as it is set by the compound’s gas/particle partitioning constant K(p) through the compound’s vapour pressure. For a largely non-volatile compound such as benzo[a]pyrene, only PDD will likely be important. For a semi-volatile compound such as nicotine, all four mechanisms can be important” *[83].

One of the main reviews focussing on EMSS notes that the particulate matter retention rate of non-smokers exposed to (cigarette) ETS is typically 11–59% [73].

#### In Situ Measurements

In a study led in 40 restaurants or cafés in Syria, the average level of RSP 2.5 was 464 μg/m^3^ [85]. Average concentrations of PM 2.5 measured the same way in Egypt varied from 56.5 μg/m^3^ to 141.6 μg/m^3^. As in the previous case, it must be understood that hookah and cigarette smoke were intermixed [86]. In a US college campus hookah lounge, measurements of PM 2.5 concentration levels at two different dates were found to be 1.1 and 2.7 times higher than the National Ambient Air Quality Standard (NAAQS) for 24 hours (35 μg/m^3^) [87].

#### Comparison with Cigarettes

A US-Syrian study compared particle concentrations (PM_2.5_, PM_10_) in ETS produced by 20 narghile smokers and 20 cigarette smokers in a poorly ventilated laboratory. It was found that “mean PM_2.5_ rose 447% for water pipe (from 48 μg /m^3^ background to 264 μg /m^3^ smoking), and by 501% for cigarettes (from 44 μg /m^3^ to 267 μg /m^3^), whereas mean PM_10_ rose by 563% for water pipe (from 55 μg /m^3^ to 365 μg /m^3^), and by 447% for cigarettes (from 52 μg /m^3^ to 287 μg /m^3^) [88]. Other results of the experiment confirm the fact a hookah does not generate SSS (thereafter called “smouldering levels”): “Mean PM2.5 and PM10 smouldering levels did not differ from background for water pipe but were significantly higher for cigarettes (PM_2.5_: 33–190 μg /m^3^; PM10: 42–220 μg /m^3^)”. Several biases have been identified in this study. The first bias is that the researchers use the word “cigarettes” in the plural form, while the given concentration is for only one cigarette smoked between 7 and 9 minutes and whereas the hookah was used (or kept lit during the measurements) over 30–35 minutes. Consequently, it appears that hookah smoke in this experiment was in fact 6.4 and 3.5 times less concentrated than cigarette smoke in, respectively, PM_10_ and PM_2.5_. A second bias is reflected in an amazing comment which states that the cigarette used in the experiment was a “Gauloise Light” because it would be “the most common cigarette used by study subjects”(sic)... In fact, particle concentrations in light cigarettes are known to be much lower than in ordinary ones. For instance, commenting on the work of McCusker, Bernstein noted that “occlusion of ventilating holes on the filters of ultra-low-tar cigarettes Barclay and Carlton markedly increased particle concentration [61,89]. The third and most serious bias is that a comparison has been made between cigarette and hookah ETS concentrations when what has actually been measured is cigarette SSS to hookah EMSS. An objective comparison would require comparing, discriminatively, and on one hand, the concentrations of cigarette SSS to that of hookah SSS and, on the other, that of cigarette EMSS to that of hookah EMSS.

When all these facts are taken into consideration, one realises that hookah smoke is much less concentrated in particles than that of cigarettes. As for qualitative aspects, it is recalled that the chemistry of hookah smoke is completely different from that of cigarette [25]. If one takes also into account the high proportion of glycerol and water in hookah smoke, it appears that it makes absolutely no sense to compare directly both smokes [7].

#### UFP (Ultra-Fine Particles)

Ultrafine particles (< 0.10 μm in diameter) are also called nanoparticles. They are present in great number in polluted urban air and therefore present a potential health risk. Their total deposition increases with a decrease of particle size and with breathing patterns of longer respiratory time. A differential lung dose may entail a differential health risk for men vs. women [90]. Indeed, because of their size, nanoparticles can easily cross the cellular membrane [84]. Anderson *et al.* state that their large surface area facilitates adsorption and delivery of potentially toxic gases to the lung [91]. These last researchers were the first to specifically examine UFP in tobacco smoke and reported a count median diameter of 0.09 μm [61].

#### Swiss Experiment

In an experiment in Switzerland (Monn *et al.*), the researchers have found that there would be 74.4 10^9^ UFP (range: 0.02 to 1 μm; median diameter: 0.04 μm) in one 1,000 mL “water pipe” (machine drawn) “breath” (i.e. a puff) and 9.24 10^9^ UFP in a single 45 mL cigarette (id.) “breath” [92]. It can be inferred that for a reference volume of 500 mL, a single cigarette actually delivers 9.24 × (500/45) = 9.24 × 11.1 = 102.6 10^9^ UFP. For the same volume, a hookah actually delivers 74.4 × (500/1,000) = 37.2 10^9^ UFP. As a consequence, the concentration of UFP in cigarette smoke equals 102.6/37.2 = 2.76 that in hookah smoke. This experiment was based on a smoking machine supposed to reflect “real” hookah smoking. However, and amazingly, only 8 grams of the smoking mixture (moassel/tobamel) were used for a 50 minute (machine) smoking “session”. Also, while the laboratory experience refers to exactly the same smoking product used in a similar system in Lebanon, the authors surprisingly acknowledge the existence of “some important differences in the breathing patterns” (tidal volume: 0.53 L vs. 1 L; puff duration: 2.6 s vs. 5s; inter-puff duration: 17s vs. 25s) between Middle East smokers and theirs. This kind of anthropometrics is amazing as, for a given product and the same configuration, Middle East narghile users smoke the same way as others in Europe and their lungs react exactly the same way to smoke stimuli… Such differences in results should be ascribed to artefacts generated by the use of the smoking machines themselves [21].

#### French Experiment

In a recent experimental study involving an artificial lung (model), Becquemin *et al.* defend the same erroneous theory that there would be a “Middle-Eastern” way of smoking vs. a “Western” one [93]. Their results are summarised in Table 1.

There are remarkable differences between the French and Swiss experiments. The post-bubbling MSS mean diameters and the concentrations are, respectively: 0.27 μm vs. 0.04 μm and 3.14 10^6^ vs. 74.4 10^6^. Besides, the French study contains other serious errors and does not present data on a key parameter: the time interval between each puffing cycle.

#### US Experiment

A US team has used a non-invasive technique to measure the particle deposition of fine particles (0.1 < diameter < 4.0 μm) and UFP in the respiratory tract of cigarette smokers, avoiding the use of sample collection bags. They found that “particle clearance, or the time it took for particle concentrations to return to baseline, was less than one minute for all particle sizes”. They also found that *“the distribution of particles in exhaled breath generally showed two maxima: one at 0.007 μm and one at 0.15 μm. The number of particles corresponding to the lowest measurable cutpoint, 0.007 μm, is at least a factor of ten greater than that measured for the next five larger cutpoints, 0.027–0.26 μm”.* Further to extraction of substrates and quantification of carcinogens from the fine particles and UFP, from the inhaled and exhaled breath of smokers, they found that *“ultra-fine particles (<0.38 μm) in the exhaled breath of smokers show measurable nicotine (125–1,200 pg/m^3^* *), cotinine (6–9 μg/m^3^), NNN (#0.3 μg/m^3^), and NNK (#0.2 μg/m^3^)”* [94].

#### Particle Growth

A striking phenomenon in this field of research is particle growth. Commenting on previous work led by McCusker, Bernstein points out that *“smoke particles double in diameter when allowed to coagulate for 30 s at low humidity. However, when smoke was allowed to coagulate for even 5 s and then humidified, a 400% increase in size was seen”. “Smoke particles from all cigarettes were less than 0.6μm median aerodynamic diameter, and particle size was not affected by filters. The commercial filters reduced particle number concentration by 20–96%, and the particle number per puff increased as the cigarette shortened. Filters reduce the concentration of cigarette smoke, but do not trap a selected size range of particles”*[61,89,95]. Results were summarised in Table 2.

The authors of another major review note that: *“Based on size and the behavior of other aerosols, only about 20% of fresh mainstream smoke entering the respiratory tract would be expected to be retained. The observed retentions of 60–80% are due to the growth of the smoke aerosol particles by water absorption in the humid environment of the lung, and the subsequent deposition of the larger aerosol particles. Typically, the smoke particles grow such that their mass is increased about 5 times and their diameter by about 70%”* [73]. Richardson is also cited in the same review for having used a “model lung” showing that the highly humid environment of the lung would let (cigarette) smoke particles grow by water condensation and Jones confirmed this finding [96,97]. As for glycerol, whose presence is of utmost importance in the case of hookah smoke, no conclusion can be drawn so far as this substance could have a dual effect [73]. It is interesting that Table 1 does not show an even greater effect of the growth phenomenon given that the smoke goes through the water but is also, subsequently, exposed to the smoker’s large lung surface area. An explanation could be that the fresh MSS smoke and simulated EMSS were immediately sampled. In the real world, and as emphasised previously (introduction), the hookah smoker, who is not a robot, lets the smoke bubble through water and also build up above the surface between two puffs. The interval between two puffs is actually variable. This way, smoke ages and its particles probably undergo a certain growth given the humidity of the environment (probably also enhanced by the heating of carbohydrates of the peculiar smoking mixture).

### 2.4. Studies on Specific Chemicals

Preliminary note: since no hookah EMSS study, specific to any of the chemicals reviewed below, was identified, the presentation is limited to results from the literature on cigarettes which allows to make relevant inferences.

#### Nicotine

In cigarette ETS, most of the nicotine leaves the particulate phase and becomes part of the gaseous phase. The intake of nicotine resulting from exposure to ETS over time reflects that to other constituents of ETS [71]. In MSS, nicotine would be predominantly (>99%) in the particulate phase [71,83]. An aforementioned US team found that UFP <0.38 μ,m in the exhaled breath of smokers showed measurable levels of nicotine (125–1,200 μg/m^3^) and cotinine (6–9 μg/m^3^) [94]. The total retention rate during smoke inhalation ranges between 90 and 100% [73]. Nicotine is extremely soluble and highly extracted from ETS within the respiratory tree [71,98]. The retention rates among non-smoking subjects exposed to ETS are typically 71–81% for this alkaloid [73]. A recent study involving a new method for estimating the retention in the respiratory tract of smokers found mean retentions of nicotine greater than 98% [99]. In a double experimental study, the uptake of tobacco smoke constituents from MSS gaseous and particulate phases, inhaled by smokers and breathed-in by non-smokers was investigated. The active smoking (20 cig./day)/passive smoking (8 h/day) ratio of nicotine varied between 75 and 90 (7.5mg-30 mg / 0.08 mg-0.4 mg) [Table 3] [100].

The amounts excreted in urine would be much lower than is found in smokers and at very high exposure, there would be no effect on blood pressure or pulse rate. Cases of headache and nausea among non-smokers have been reported only under conditions of heavy exposure [74]. This weak effect of nicotine was also confirmed by other researchers [101].

#### Carbon Monoxide

Carbon monoxide increases heart rate. This gas binds to haemoglobin, myoglobin and cytochroms. Carboxyhaemoglobin levels are generally high, particularly among jurâk smokers [1]. However, the diverse types of charcoal, tobacco-based mixtures, the size of the device play an important role in variations [102]. Patrons who spend several hours in ill-ventilated hookah lounges often feel numbed and suffer from headaches. CO seems to be directly involved in the vascular complications related to smoked tobacco as opposed to other ways of using it, e.g. smokeless tobacco of the Swedish SNUS type [1,103]. The total retention, during cigarette smoke inhalation, in the human respiratory tract is of the order of 55–65% during cigarette smoke inhalation [73]. This is confirmed by a recent study involving a new method for estimating the retention in the respiratory tract of smokers which found an even greater average retention of CO: 79% [99]. As a biomarker of ETS, CO has a poor sensitivity and specificity. Beside environmental sources, CO is also produced by endogenous metabolism and only small changes in CO have been reported after ETS exposure [71]. These last facts are confirmed by several studies on hookah smoking carried out in cafes/hookah lounges in Lebanon, France and the USA [1,14,104].

Three decades ago, Russell *et al.* carried out a famous artificial experiment in which 20 volunteers spent about 78 minutes seated in an unventilated smoke-filled room of about 43 c.m. (15x12x8 ft). Eighty cigarettes and two cigars were burnt or smoked. The average ambient CO concentration reached 38 ppm. Thanks to COHb monitoring, the researchers concluded that “the amount of inhaled CO by non-smokers as a result of their exposure to ETS was about the same as would be expected if they had actively smoked and inhaled one cigarette”[105]. Other researchers have performed a more realistic although “acute natural exposure” experiment in which seven non-smokers were exposed to tobacco smoke under natural conditions for two hours in a public house. They found that the increase in expired CO of 5.9 ppm was similar to increases in smokers after a single cigarette [101]. Scherer *et al.* measured an active/passive ratio of CO varying between 2.7 and 4.2 (40 mg-400 mg / 14.4 mg-96 mg) [Table 3] [100].

Rylander *et al.* state that under realistic environmental conditions, CO concentrations reach about 10 ppm and that the higher concentrations that may be reported here and there would represent “only transient values or levels reached under experimental conditions”. They add that “if the exposure to 10 ppm were to prevail for 8 hours, the resulting COHb concentration would be 1.9%”, still below the WHO limit of 4% [74].

In fact, early studies have shown that if the CO concentration reached 3 to 4% in cigarette MSS, 6% in cigar smoke and 2% in pipe smoke, it would be *a priori* fatal for health to stay in such an environment. The reason is that smoke is diluted to a great extent and is breathed in only once out of 12 to 15 inspirations. As a consequence, CO intake would be moderated: about 15 to 20 mL for a cigarette and 50 to 100 mL for a cigar. It was noted that, for this reason, non-inhaling smokers have CoHb levels barely greater than non-smokers. Those who inhale would reach 5 to 7% (15–20 cigarettes) and 9 to 13% (25 cigarettes and more) [106].

#### CO Intoxication (Cigarette and Hookah)

In Saudi Arabia, only one shisha smoker out of 24 cases of CO-related intoxications of diverse types could be identified [107]. Recently, two cases of hookah CO-intoxication were widely advertised in the French media [108]. The original report was used as scientific evidence to support a stringent ban on hookah lounges in this country. Unfortunately, the brief document did not provide any data supporting the hypothesis of the existence of a large-scale problem. Indeed, about 1,000 hookah lounges were identified in France, no other similar cases have been reported. This is also true in other parts of the world. Consequently, what this report shows is that prevention should have focussed on the long overdue message that hookah lounges must not be ill-ventilated [1]. From a recent study from Jordan involving a large sample (14,310 subjects), conclusions can be drawn that support this last public health position. The study showed that the increase in arterial blood pressure in “pure”/exclusive shisha smokers varied from 92.57 ±13.90 to 92.62±10.58. The heart rate changed from 76.40±10.46 to 76.81±10.19. This is in agreement with another previous study in this country whereby researchers reported a slight increase in the above parameters [109,110]. Certainly, this was active smoking, not exposure to ETS. However, and since the active smoker is the first victim of her/his own passive smoking [13], this brings out the importance of ventilation. Indeed, cafes in the Asia and Africa in general, and in the Middle East and Jordan in particular, are usually efficiently ventilated, contrary to their counterparts in other parts of the world [1].

In the Swiss experiment described previously, not only UFP in MSS were measured but also CO concentration. It is interesting because it allows a comparison between cigarette and hookah concentrations. It was found 1.79 mg CO for a 1000 mL hookah (machine) puff and 1.06 mg for a 45 mL cigarette puff [92]. Therefore, for a common reference volume of 500 mL, a cigarette actually delivers 1.06 × (500/45) = 1.06 × 11.1=11.76 mg CO. For the same volume, a hookah actually delivers 1.79 × (500/1,000)= 0.89 mg. Noting that one machine-smoked cigarette produces about 500 mL, that is 11 puffs of 45 mL, it can be inferred that CO concentration in cigarette smoke equals 11.76/0.89= 13.21. Therefore, hookah smoke may be 13 times less concentrated in CO than cigarette smoke. This result also offers an explanation to the existence of so many stuffy hookah lounges packed with patrons and full of smoke for hours on end.

#### Carbonyls (Aldehydes)

The focus will be on aldehydes and particularly acrolein and formaldehyde. These are biological agents which cross-link proteins. They also stimulate mucus secretion in the airways [74]. The Tobacco Free Initiative of WHO warns against carcinogenic chemicals to be found in ETS. Among them, acetaldehyde, acrolein and formaldehyde are listed: “when inhaled, these poisons are concentrated and quickly spread throughout the body, leading to a range of serious diseases” [35]. Aldehydes are normally found in the gaseous phase of the smoke except for formaldehyde which may also be found in the particulate phase, probably because it is highly soluble in its water fraction. During cigarette smoke inhalation, the total retention of aldehydes in the human respiratory tract is approximately 90%. Mouth retentions are also high (>30%) for these compounds and average lung retention for some are >95% [73,111]. A recent experimental study involving human subjects offers a quantitative evaluation of carbonyl levels [of formaldehyde, acetaldehyde, acrolein, propionaldehyde, crotonaldehyde and *n*-butyraldehyde, and of two ketones (acetone and 2-butanone)] in EMSS. A high retention of all carbonyls was found: above 95% for aldehydes. Ketones were retained to a lesser degree. Retention of acetaldehyde would be in very good agreement with pre-existing literature on this issue (cited: Dalhamn *et al.* 1968a, b; Laskowski 1951) [58]. This is confirmed by a recent study involving a new method for estimating the retention in the respiratory tract of smokers which found an average retention of acetaldehyde of 99% [99]. Scherer *et al.* measured an active/passive ratio of formaldehyde varying between 4 and 5 (0.4 mg-1.8 mg / 0.08 mg-0.4 mg) [Table 3] [100].

#### Recent and Older Studies on Aldehydes in Hookah MSS

Average yields of formaldehyde, acetaldehyde, acrolein, propionaldehyde and methacrolein obtained with a smoking machine and involving moassel/tobamel were elevated: 630, 2,520, 892, 403, and 106 μg, respectively, per smoking session [112]. A major source of some aldehydes are sugars, particularly important in moassel/tobamel. Concerning formaldehyde, its MSS yields from cigarettes containing sugars were significantly higher than that from a control cigarette [73]. The hard and unrealistic parameters of the above-mentioned smoking machine (one 530 mL puff every 17 s for a full hour) may also explain the high yields for this aldehyde. As for acrolein, one may also wonder, in view of such hard parameters, whether or not a part of the yield is a result of the cracking of glycerol. Also, an intense machine smoking regime can make water become quickly saturated and therefore chemically stripped of its natural obstructing properties.

Interestingly, in the case of tumbak (plain moistened tobacco with no added sugars), the product traditionally smoked over the past centuries in Asia and Africa, previous studies do not mention high levels of aldehydes in MSS, but rather emphasise their water solubility. For instance, Guillerm and colleagues, who early investigated the compounds playing a role in cilia toxicity, found that they were water-soluble and identified two major ones: acrolein and formaldehyde. They speculated that their water solubility could be an explanation for the widely used narghile by Middle Eastern populations in spite of the great amounts of tobacco consumed in this device. They relevantly noted that narghile makes the smoke less irritating [113]. Huber and colleagues found that a very small physiologically wetted surface was capable of complete detoxification of, among other cytotoxins, acrolein and acetaldehyde [114].

### 2.5. Other Substances (PAHs, Phenols, Benzene, Toluene, NOx, Heavy Metals, etc.)

#### PAHs (Polycyclic Aromatic Hydrocarbons)

PAHs are the result of incomplete combustion and can be found virtually anywhere in the environment. Some of them are powerful carcinogens. Interestingly, a comprehensive review of Indian studies related to smoking reports that in areas where biomass fuels are used for cooking, exposure to benzo[a]pyrene would be equivalent to smoking about 20 packs of cigarettes per day [115]. PAHs can be found in the air, in the workplace, food, ETS and coal-tar-containing medications [36]. In a hookah, the main (if not the only) source of them appears to be the charcoal used to heat the smoking mixture. Indeed, a team in Saudi Arabia did not identify any of them when using an electrical system [116]. Scherer *et al.* measured an active/passive ratio of benzo[a]pyrene varying between 70 and 150 (0.15 μg -0.75 μg / 0.001 μg-0.011 μg) [Table 3] [100]. Moldoveanu tested the retention efficiency of 20 PAHs. The results show that PAHs with a molecular weight lower than about 170 Daltons are retained with high efficiency. The heavier molecules are less retained, but even compounds such as indeno[1,2,3-cd]pyrene, dibenz[a,h]anthracene, and benzoperylene are retained with efficiencies around 50% “[65].

#### Phenols

A historic study by Hoffmann et al. found that a hookah could filter up to 90% of phenols present in MSS [117]. Baker and Dixon cite Ingebrethsen, who reported an average retention rate (cigarette EMSS/MSS) of 100% for phenols [62,73]. Moldoveanu *et al.* tested the retention efficiency of 20 hydroxybenzenes (phenols) from MSS and found that phenols were retained with high efficiency: typically above 80%. Interestingly, the researchers note that the high retention of this class of compounds was expected since phenols are polar compounds with relatively low molecular weights between 94 (for phenol) and 152 (for a propyldihydroxybenzene) [67]. This interpretation would also explain Hoffmann *et al.*’s above mentioned results since the water inside the hookah may offer a similar polar environment to that of the respiratory tract.

#### Benzene and Toluene

The major sources of benzene in outdoor ambient air are emissions from traffic exhausts. Benzene is classified as a human carcinogen [36]. Baker and Dixon cite Backhurst and Martin (1973) who reported a total retention average of 75% for both moderate and deep inhalation of benzene. As for toluene, they cite Dalhamn *et al.* (1968) who reported an average retention rate of 93% [73]. Toluene, being insoluble in water, would be retained “in those regions of the lungs where there is surfactant, e.g., the alveolar epithelium. The lipophilic nature of the surfactant-coated epithelial surface probably contributes to the retention of the lipophilic constituents of complex aerosols such as tobacco smoke” [111]. Scherer *et al.* measured an active/passive ratio of benzene varying between 3 and 5 (200 μg -1200 μg / 40 μg - 400 μg) [Table 3] [100]. Moldoveanu *et al.* tested the retention efficiency of benzene and toluene from MSS. They showed that benzene was retained by 89% to 98%, and toluene in similar proportions (87% to 99%) [66].

#### Nitric Oxides

Nitrogen oxides damage cell membranes in the lung and also combine with amines to produce nitrosamines which are oxidized to alkylating agents (HN_2_). These are both potent carcinogens and sensitising agents [74]. Nitric oxide is to be found entirely in the gas phase. Cigarette MSS total retention of nitric oxide in the human respiratory tract is of the order of 100% [73]. NO is selectively taken up by the alveolar pulmonary capillaries. NO, like CO, would not be taken up by the airways. However, on contact with the alveolar capillaries it is taken up 4.5 times faster than carbon monoxide. The solubility of NO in water is greater than oxygen or CO but it is still very low [111].

#### Heavy Metals

Considering, not only active smoking but even passive smoking, it was early recalled that WHO TobReg warned that “second-hand smoke from waterpipes [...] poses a serious risk for non-smokers” [55]. Interestingly, the underpinning reference states that “the higher content of heavy metals in waterpipe smoke compared to cigarettes may also have adverse health effects on exposed non-smokers” [56]. However, no study on heavy metals in hookah ETS has been identified so far, at least for those for which concern was raised: namely lead, chromium, cobalt, nickel, beryllium and arsenic. As for the source of heavy metals that could be found in hookah MSSS, it is doubtful and results from different studies are contradictory [4]. Indeed, in Saudi Arabia, researchers determined by atomic absorption that out of 14.685 mg (heavy) metals present in 1 g of the jurak paste, only 3.075 μg were transferred to the smoker [116]. In an unpublished report about an experiment conducted in Russia, arsenic was not detected. As for the potential intake by exposed non-smokers, the case of cadmium is more documented. Scherer *et al.* measured an active/passive ratio of cadmium varying between 110 and 1,500 (1.5 μg / 0.001 μg-0.014 μg) [Table 3][100]. In India, an association was found between tobacco smoking habits of male and female rural subjects using hookah and increased Cd levels in hair and nails [118]. However, possible confounding factors (simultaneous use of cigarettes or bidis, pollution, diet, *etc.*) were raised [6].

#### Nitrosamines

Scherer *et al.* measured an active/passive ratio of tobacco-specific nitrosamines varying between 2,300 and 4,500 (4.5 μg - 45 μg / 0.002 μg - 0.010 μg) [Table 3][100]. A study previously cited about nicotine found that UFP <0.38 μm in EMSS showed measurable NNN (#0.3 μg/m^3^) and NNK (#0.2 μg/m^3^) [94]. A recent study involving a new method for estimating the retention in the respiratory tract of smokers found mean retentions of two tobacco-specific nitrosamines significantly higher for deep inhalers (84% for NNK and 97% for NNN) than those for normal inhalers (63% for NNK and 84% for NNN) [99]. Interestingly, the title of a comment on this last study states that “NNK is not insoluble in water” [119].

#### Acetone and 2-butanone

Acetone is normally retained in the range of 90% to 95%. The retention for 2-butanone would be slightly less absorbed than aldehydes, with an average retention around 95% [58]. Baker and Dixon cite Dalhamn *et al.* who reported an average retention rate of 86% for acetone [73].

#### Radiotoxic Elements

Only one study has identified so far the potential hazards of inhaling hookah MSS. Tobacco, including the hookah tobacco component, contains minute amounts of radiotoxic elements such as (210)Pb, (210)Po and uranium. It was found that the average concentrations of natural radionuclides in moassel tobacco pastes from Egypt and Saudi Arabia are comparable to their concentration in Greek cigarettes and tobacco leaves, and lower than that of Brazilian tobacco leaves [7].

#### Miscellanea

A recent study involving a new method for estimating the retention in the respiratory tract of smokers found an average retention of ethylene of 33% [99]. No relevant study was found about the retention rate of hydrazine and HCN, two highly toxic substances. However, since both of them are miscible with water, one can expect a high retention rate either in the water of the hookah or in the highly humid respiratory tract environment. Baker and Dixon cite Ingebrethsen, who reported an average retention rate of 92%, 99% and 87% for hydroquinone, triacetin and glycerol, respectively [73]. The case of glycerol is discussed below. Dalhamn *et al.* are cited for having reported an average retention rate of 91% and 99% for acetonitrile and isoprene, respectively. As for Lehmann, he has found an average retention rate of 70% and 92% for ammonia and pyridine, respectively [73]. Feng *et al.* found an average retention of isoprene of 52% [99]. Water, particularly that contained in the base of the hookah, may act as an anti-oxidant against a category of short half-life free radicals [120]. Sulphur dioxide is soluble and may either be retained in the water of the hookah or the respiratory tract. Of course, there are many other substances and (historic) details about them can be found in the reference review [73].

### 2.6. Further Discussion

Considering tobacco products in general and hookah in particular, the issues of water solubility of smoke constituents, glycerol as an important ingredient, ventilation, ageing of smoke, smoke dilution, hookah lounges, pregnant women and children, are discussed below.

#### Effect of Water Solubility of Smoke Constituents

Experts have noted that the main acute effects of ETS would be eye irritation and bronchial irritation and these would be caused by the particulate matter and certain gases in the smoke, such as formaldehyde, acrolein and ammonia [74]. In a hookah, the smoke meets a first water environment featured by the water in the vase and then another highly humid environment: the respiratory tract. The water solubility of the above substances and the corresponding retention rates reviewed above may explain why such effects in non-smokers exposed to hookah smoke are absent and, by the same token, confirm the traditional social acceptability of hookah smoking. An experimental study involving human subjects, and already cited, evaluated the retention of 160 compounds from cigarette MSS. The authors concluded that about one third of the evaluated compounds -including molecules with lower molecular weight and relatively good solubility in water- were retained by more than 66%” [68]. Baker and Dixon cite Ingebrethsen who suggested that the order of decreasing deposition for constituents (these were: phenol > nicotine > triacetin > propylene glycol > 3-hydroxypyridine > neophytadiene > hydroquinone > glycerol) would approximately correspond to the order of decreasing volatility although other factors such as water solubility would also likely be involved [73]. Feng *et al.* found that the more soluble compounds might have higher retentions. The water solubility (pH of 7.4) of three particulate phase constituents was in the order of nicotine > NNN > NNK. Respiratory retentions of the gas/vapour phase constituents may be associated with their solubility as well. The decreasing order of retention was found to be acetaldehyde > isoprene > ethylene, a finding in agreement with the blood solubility of these three compounds [99]. Higgenbottam *et al.* have noted that the water-soluble acetaldehydes are retained in the aqueous linings of the mouth and that the water-insoluble toluene is retained in those regions of the lungs where there is surfactant, e.g., the alveolar epithelium. They explain this phenomenon by the fact that the lipophilic nature of the surfactant coated epithelial surface probably contributes to the retention of the lipophilic constituents of complex aerosols such as tobacco smoke [111].

#### Glycerol

Glycerol, present in the smoking mixture, forms, with water, a great proportion of hookah MSS. Probably, the same proportions are found in glycerol cigarettes in which tobacco is heated and not burnt as in the hookah. Researchers have noted, about these cigarettes, that the smoke particles in which nicotine is transported are comprised mainly of glycerol and water rather than tar and that glycerol is harmless, easily absorbed, and metabolised as a source of energy [121]. Glycerol would be retained by 87% according to Ingebrethsen [73]. On an aerodynamical level, glycerol particles inhaled via MSS are thought to pick up water vapour more readily than smoke particles without glycerol. However, the picture may not be so simple and an experiment by Hickey and Martonen has shown that its presence reduced the growth of hygroscopic aerosols produced by nebulisers in a lung system [73]. This needs further clarification.

#### Ventilation

Rylander *et al.* emphasised that any estimation of exposure levels of ETS requires that the following parameters be known: number of cigarettes, cigars and pipes smoked/volume room air; ventilation characteristics (air changes/unit time) [74]. However, for anti-smoking researchers, ventilation does not eliminate all risks [122]. During the measurement of CO levels, in French hookah lounges in 1998, ambient levels of CO were found to vary between 10 and 60 ppm, according to place, ventilation, number of lit hookahs, presence of cigarette smokers, *etc.* However, it was reported that the smoke of cigarettes intermixed with that emitted by hookahs which also entailed the use of embers known to generate high levels of CO [2]. Since the ban on indoor-smoking in France (02 Jan. 2008), hookah lounges stayed open and, given that cigarettes are not allowed, it is worth noting that the CO levels have significantly dropped, although studies are needed. The issue of ventilation has been discussed by researchers who oppose too stringent laws. It was recalled, for instance, that at one time or another, smokers or non-smokers generally take some action (e.g. go away from the smoking area, open a window, *etc.*) to reduce the pollution level [74]. Unfortunately, these important facts have, too often, been glossed over in many studies.

#### Ageing of Smoke

Many studies on cigarette ETS have glossed over this point of utmost importance, particularly in hookahs. For instance, Borgerding has noted that *“decrease of NO in the fresh smoke is accompanied by an increase of NO_2_. NO_2_* *concentration reaches a maximum after about 1 min and then it decreases. CH_3_OH concentration in the smoke is stable for about 10 s, after which it decreases parallel to the NO_2_* *concentration. That means there will be a reaction between the nitrogen oxides and CH_3_OH, resulting in artificial formation of methyl-nitrite CH_3_NO_2_, a component which is not present in fresh tobacco smoke”* [59]. This is one of the reasons why cigarette ETS is different from mainstream smoke. Indeed, while ageing, smoke undergoes numerous transformations which add to its complexity. Perhaps it would be relevant to recall that such a passionate debate over ETS toxicity concerns a smoking instrument (the cigarette) whose length is about 10 cm and that the above example is only one among other facts which are usually not taken into consideration. In these conditions, conclusions about cigarette ETS toxicity, regardless of their soundness, should not be considered as equally valid for hookah smoke. Cigarette and hookah MSS are different and, for other reasons also (temperatures, no SSS is generated by hookah, inter-puff time, *etc.*), it was shown that such an inference was not supported by the available literature [25,29]. Furthermore, the smoke (MSS) inside a hookah runs a distance of about 250 cm from its production site (the bowl) to the mouth of the smoker. It is above all a product of a distillation process and it has to bubble through the water filter. Then, it builds up above the surface of the latter between two puffs. At the end, what the smoker, most of the time, inhales (when it does), apart from the first puff (which will be diluted with more air than the following ones), is not fresh but aged smoke, very aged smoke. Indeed, when presenting the differences between ETS and MSS in the introduction, it was already noted how, in the 10 cm tobacco rod of a cigarette, particles may build up in the void volume of the cigarette during the 1 minute smoulder period between two puffs.

#### Dilution

This point is also very important and some facts are even counterintuitive; for instance that lung cancer rates were found higher in non-inhalers than in heavy smokers because of “subtle interactions between the amounts smoked, the tar/nicotine yield of the cigarette, and the style of smoking” [123]. A cigarette smoker typically generates a smoke puff volume of about 50 ml which is diluted with air 10 to 20 fold when inhaled [114,124]. In an early publication, commented upon by Bernstein, Higenbottam *et al.* proposed that the usual pattern of smoking consists of an initial drag of smoke into the mouth followed, after a variable pause, by a subsequent inhalation of smoke into the lungs. This could minimise the irritant qualities of the tobacco smoke [61]. In the case of hookah smoking using moassel/tobamel (not tumbak or jurak), it was noted that the users feel that the smoke is very mild – particularly because of the actual water trapping of notable irritants. One direct consequence is that they often inhale considerable amounts of the smoke: randomly varying between 100 mL at least (but less sometimes) and up to 500 mL and sometimes more. These undiluted quantities of smoke go directly into their lungs with no previous stocking inside the mouth as [1]. This phenomenon may sometimes be observed in some cigarette smokers. Citing Tobin and Sackner (1982) who used a non-intrusive technique (inductive plethysmography), Bernstein reports that the inhaled (in two phases) volumes ranged from 270 to 1,990 mL [61]. This fact can explain why modern hookah smokers inhale clouds of smoke. They simply do not dilute smoke.

#### The Case of the Hookah Lounges

For almost two decades and for many reasons analysed elsewhere, the traditional Middle Eastern coffee house has become a sort of model that was “exported” all over the world thanks to the globalisation process of the 1990s (Table 4). Today, the most appropriate sociological description for this kind of “hospitality venue” would be neo-orientalist cafe; not only in the USA or Europe but also in the Middle East itself, i.e. the “Orient” in which the original café was invented at the beginning of the 16^th^ century. A few decades later, it would adopt the narghile which became its distinctive trait when compared to the European café model. This way, the hookah has not only been a discovery for some people but also represents a revival of an existing century-old tradition in Asia and Africa. Traditional hookah smoking is generally performed in the open air as early health-oriented socio-anthropological observations show [5]. A survey in Syria among 1,118 non-smokers showed that there were 48.6% households with 1 cigarette smoker or more and only 4.2% with 1 narghile smoker or more [76]. Studies on exhaled CO in non-smokers exposed to hookah ETS were conducted in cafes and hookah lounges (Lebanon, France, USA). They did not show any substantial change [1,14,104]. Therefore, the only problem posed by these places may be active smoking in sometimes ill-ventilated conditions. However, it must be borne in mind that these places are mainly patronised by individuals who are aware that they going to a place dedicated for smoking.

#### Pregnant Women and Children

Problems related to the existing studies on children exposure to hookah smoke were discussed under subsection “Epidemiological Studies approaching Hookah ETS” [28,63,64]. Other recent studies have raised the potential hazards of hookah ETS for pregnant women. In Iran, concern was recently raised over the prevalence of smoking local water pipes (qalyân, narghile) among pregnant women in Southern Iran [125]. The researchers found that exposure to non-cigarette ETS reached 11.5%. Also, considering active smoking in pregnant women as a form of ETS to which the foetus is exposed, the WHO EMRO report cites a study (led by Abdella) on sleep breathing disorders in cigarette and goza smokers. Goza is a small local Egyptian water pipe, different from the shisha. By contrast with the latter, the charcoal is in direct contact with the smoking mixture. Such a smoking mode, according to the above study, would have odds of 2.94 (CI: 1.08–8.06) of being associated with an apnea/hypopnea index above 5 than in cigarette smokers. From there, and with no preliminary discussion on the exact career of the smokers (simultaneous use of cigarettes, former cigarette smokers, *etc*.), the WHO experts amazingly speculate: “This may lead to a query: does ETS from water pipes have more hypoxic effects on infants than has been shown with ETS from cigarette smoking?” [28]. Perhaps it should be noted that goza smoking, unlike shisha, is overwhelmingly a male habit.

A study often cited in this field found that the adjusted odds ratio of having babies with low birth weight among narghile smokers was 1.89 (CI: 0.67–5.38). The risk increased to 2.62 (CI: 0.90–7.66) among those who started smoking narghile in the first trimester. A stronger association and a dose-response relation were found among cigarette smokers. Apgar score and respiratory distress were also noticeable [126]. In fact, apart from the fact that the career of the participants was very vague, there was a first bias -acknowledged by the authors themselves- because those who stopped smoking before getting pregnant were considered as “non-smokers”. Indeed, an ex-smoker cannot be classified as such [13]. Yet, the authors were very cautious, stating that “most importantly, it is rather difficult to measure the pure effect of narghile smoking because most narghile smokers are current or former cigarette smokers”. They also stressed the importance of considering the type of tobacco-based smoking mixture (tumbak or moassel), its amount, the time spent, the hose length, the amount, *etc.* As they noted, this could affect CO concentration in blood. Unfortunately, these observations were, most of the time, glossed over by those who cited this important study. Furthermore, and as this review shows, the nature of hookah smoke, and particularly ETS, is very different from that generated by cigarettes. An animal experimental study from Saudi Arabia showed that prenatal exposure to shisha smoke lowers the response to novel environments whereas passive exposure to the smoke of the local pipe (shisha) during pregnancy had no effects on the gestational period, number of pups, birth weight, and body weight growth [127]. However, pregnant women should refrain from active smoking and particularly hookah.

### 3. Methods

This review on hookah ETS builds upon an uninterrupted and ongoing biomedical and anthropological research work, including reviews and updates–as stated in the introduction-, initiated one decade ago [5,8].

#### Eligibility Criteria

The eligibility of the publications cited and discussed here was assessed based on the author’s 13 year pioneering experience in this field. The selected materials had to be peer-reviewed and published in scientific journals. They had to offer relevant analysis of the chemical composition of EMSS and its hazards for non-smokers.

#### Search Strategy

The full text of hundreds of biomedical journals -thanks to such online tools as ScienceDirect and Springer (publisher)- and the main electronic databases (Medline; Embase; “Web of Science”; *etc.*) were searched and regularly consulted, particularly for new publications reflecting any sudden recent interest in this issue in line with national bans on indoor smoking. The period covering the preparation of the core of the review extended from the half of January 2007 to July 2008. However, and to show the importance and sensitiveness of the issue, only recently (i.e. beyond that span of time), a new concept has emerged with the hazards posed, far beyond SHS, by cigarette Third-Hand Smoke. This has been commented upon and discussed in the conclusion as it may be relevant for the further identification of hookah ETS hazards.

Databases, among other online resources, were searched thanks to Boolean equations including “hooka[h]”, “hukka[h]”, “shisha”, “chicha”, “water-pipe”, ““waterpipe”“ (in one word), “goza”, “guza”, “narghile”, “narguile”, “cigarette”, “cigar” and “pipe”, as chief terms. These were logically linked to the following (group of) of keywords: “environmental tobacco smoke [smoking]”, “passive smoke [smoking]”, “second-hand smoke [smoking]”, “exhaled mainstream smoke”. In a last phase, the search was extended to the world wide web and particularly to “Google Scholar”, a useful tool for full-text search of academic publications in all languages, not only English. Indeed, another methodological concern was to take into account all relevant materials irrespective of their language in order to avoid another kind of frequent bias [11,12]. As an example of the latter, researchers (in an aetiological study on hookah smoking and cancer) have actually dug out scientific publications which have been ageing for decades on the shelves of libraries [8]. “Provincialism” was thus avoided to a great extent. An additional French database (Toxibase) was searched for the same purpose. Finally, the work was completed by an investigation in libraries of Asia and Africa thanks to the collaboration of a wide network of researchers in these countries. This proved useful particularly in India, Saudi Arabia and Egypt.

#### Study Selection, Data Collection and Analysis

The first listing of studies was cross-referenced with the bibliographies of the two central and historic reviews (Baker and Dixon, on one hand, and Bernstein, on the other [61,73]). This tuning process has allowed the identification of many relevant studies on human toxicants to be found in ETS and how they transfer, and to what extent, from MSS to EMSS. A striking example is the one related to studies on particle growth in humid environments. It has proved highly relevant because of the presence of water in the hookah.

The author has worked independently, scanned all abstracts and eligibility was assessed from the full text of the corresponding publications. It is noteworthy that no previous review on hookah ETS was identified. Publication bias (“cherry-picking”) was avoided by considering the actual existence of tobacco industry studies as well as those from the anti-smoking research groups including WHO-supported organisations working towards the implementation of the World Tobacco-Free Agenda (FCTC, Tobacco Free Initiative). The reviewed literature represents about one third in the former case and about two thirds in the latter. It was kept in mind that anti-smoking researchers consider that tobacco industry studies are frequently subject to bias and too often scientifically irrelevant.

If the tobacco industry studies had been brushed aside, as some anti-smoking organisations suggest, there would not have been any need to carry out the present review. Indeed, the overwhelming work on EMSS has been done by researchers working for the tobacco industry. Indeed, being anti-tobacco or pro-tobacco proves to be of no help in this field. By contrast, being “passionately prodebate and proscience” certainly does [128]. An embargo on tobacco industry studies, which are of an undeniable scientific interest, and almost the only ones on this issue (EMSS), would definitely be anti-science. As a good example, a journal like Beiträge zur Tabakforschung International [Contributions to Tobacco Research] has published only recently seminal studies on EMSS [58,65–68].

#### Limits Set on this Review

Hookah smoking is a novel and complex field of research where biomedical and human-centred anthropological considerations and interpretations compete and sometimes collide. In these conditions, the present review does not pretend to be exhaustive as far as the existing literature on cigarette ETS -with a focus on EMSS- is concerned. Yet, it appears that the full range of the present scientific knowledge relevant to hookah smoking has been covered. In the introductory section (“Overview of Landmark Studies on Cigarette ETS”), the work has focussed on the existing reviews, sometimes leaving aside interesting studies on the association between diseases and cigarette ETS. It is recalled, once again, that the issue reviewed here is hookah smoking and not cigarette smoking, EMSS and not SSS, virtually absent in the former.

### 4. Conclusions

In situations where individuals are exposed to the clouds of ETS exhaled by modern hookah smokers (using moassel/tobamel), it appears, using cigarette smoke retention models and rates from studies reviewed in the present work, that:

1. hookah smoke is made up of a large amount of glycerol and water (probably around 80% or more) and that these two substances are harmless;
2. exposed non-smokers to hookah smoke would retain in their respiratory tract 11–59% of the remaining (EMSS) particulate matter and 71–81% of nicotine;
3. exhaled CO measured in non-smokers exposed to hookah ETS in different settings (cafes, hookah lounges) and countries does not vary;
4. the respiratory tract of active hookah smokers would retain up to 95% of the main aldehydes which are known to be water soluble and, consequently, also stopped to an unknown proportion in the water vessel of the hookah.

Furthermore, it should be emphasised once again that there is a lack of sound epidemiological research on the health risks of hookah active smoking as far as long-term complications are concerned. No conclusion can be drawn from the existing studies (on pathologies like oral, gastric and bladder cancer, contact eczema, tuberculosis or aspergillosis, *etc.*) because of striking confusion factors such as the simultaneous use of other products [e.g. qat, cigarettes, bidis, *etc.* ] or a strongly neglected hygiene (hose, water not changed, *etc.*). Most of the time, the remote and recent career of smokers (former cigarette smokers having quit for a long time and suddenly indulging in hookah smoking; or cigarette smokers having “switched” to hookah smoking; *etc.*) were not given any detail [1]. All these facts and others lead to the conclusion that hookah ETS (not MSS) hazards will remain unwarranted until a study shows that minute amounts of toxicants present in hookah EMSS may cause serious diseases as some researchers state about cigarette ETS [129]. Most recently, an interesting study showed that, given that there would be no safe level of exposure to tobacco smoke, ThirdHand Smoke (defined as residual tobacco smoke contamination that remains after the cigarette is extinguished) may be extremely hazardous, particularly for children at home [130]. It is also noted that in the case of cigarette smoking, where, unlike hookah, SSS is generated, exposed non-smokers do not breathe deeply, particularly when they are exposed to tobacco smoke. This fact is of utmost importance as many statements about ETS cigarette assume a similarity of inhalation patterns between active and passive cigarette smokers. Perhaps the only problem regarding hookah ETS might be odours. Experts had once noted that “although many people dislike the smell of burning tobacco, there are some who enjoy it” and added that “this is especially true of cigar or pipe smoke, which nevertheless contain higher concentrations of irritants than cigarette smoke” [74]. In the case of heated flavoured (apple, strawberry, rose, *etc.*) tobacco-molasses mixture (moassel/tobamel), it is noteworthy that non-smokers do not feel bothered by the smoke [2]. The only problems reported so far are social nuisances caused by the smell of flavours, particularly in urban settings.

Citing a report of the Institute of Medicine (2001), a recent paper concludes that: *“While prevention and cessation is the most effective way to eliminate the health risks of cigarette smoking, the use of cigarettes and other tobacco products will continue. In spite of stringent smoking restrictions in the United States, it is expected that, in 2010, approximately 10–15% of the adult population in this country will not be willing or able to give up tobacco consumption (Institute of Medicine, 2001). For these people it is most important to develop products of harm reduction”* [26]. Informing on the hazards of active smoking (cigarette or hookah), on which there is a wide consensus, is important and accepted by the smokers themselves around the world. However, hyping ETS hazards may have backlash effects. A smokeless product of the Swedish SNUS type is probably the best and universal harm reduction tool [8]. Two decades ago, a harm reduction cigarette which heats tobacco instead of burning it, and which generates no SSS, had appeared under successive forms and names and was positively assessed by prominent world experts [121,131]. However, it met a wide opposition. Today, the market is displaying a multitude of new alternatives to cigarette and hookah smoking. For instance, recent research by the Tobacco Industry has been done on the Electrically Heated Cigarette Smoking System (EHCSS) in which tobacco is only heated during each puff and no SSS is generated. Gas-vapour phase ETS markers would be reduced by 97% and total RSP by 90% [132]. Also, switching from conventional cigarette smoking to the EHCSS would result in substantial reductions in concentrations of several ETS markers [133]. Recently, the Chinese have made their way in this open market by offering electronic cigarettes, cigars and pipes. These products contain no tobacco but vaporise nicotine and flavours. It is noteworthy that all these inventions more or less mimic the narghile principle. An E-narghile is also in project.

The scientific evidence about CO hazards connected with the hard use of hookah smoking, particularly in ill-ventilated places, was sufficient and the best public health message. Amazingly, it was dismissed and aggressive public health plans were favoured as against cigarette ETS. They may have gone too far, not realising that a confrontational approach to prevention is generating a growing reaction that psycho-sociologists explain as an attack on the very individual identity in the case of cigarette smokers [13]. In the case of hookah smoking, there is also a collective identity because of its important sociological, anthropological and historical dimensions. Perhaps it is time to put all environmental health risks in perspective as a timely book suggests [134]. Cigarette or hookah ETS is certainly a problem but not a public health one, as alcoholism is.

#### Glossary

**Bidi** (“beedi”): a tobacco product in the Indian sub-continent. “Bidis are made of crude sun-dried tobacco wrapped in a dried Tendu (Dyospyros melanoxylon) leaf” [78].

**Bowl:** Locally called “chillum”, “chilam”, “ras”, “hagar”, *etc.* The top part of the hookah, containing the smoking mixture. When the latter is tumbak, the charcoal pieces are in direct contact with this product. When it is Moassel (tobamel), a thermal screen made of a tin foil (kitchen aluminium) is inserted between both.

**Hookah:** An ancient pipe traditionally used in Africa and Asia. This word is the one used in Indian, Pakistan and many other English speaking countries. The height of this apparatus can reach 2 m and its suction hose 5 m. The modern version, i.e. the shisha, is smaller (0.75 m an 1.50 m respectively). Hookah is an Arabic word for vase, vessel (i.e. of water).

**Jurak:** A mixture of about 30% tobacco and 70% molasses/honey/glucose syrup and minced fruits. It does not contain glycerol as moassel. It is strong (nicotine), generally black and barely used outside Africa and Asia.

**Moassel:** Also called tobamel (“tob” stands for tobacco and “mel” for honey in Latin). Means “honeyed” in Arabic. A mixture of about 30% tobacco and 70% molasses/honey/glucose syrup plus glycerol and essences. It is much less stronger than jurâk (nicotine): a sort of “light” version of it. It is widely used on all continents now. More recent than jurâk, it appeared in the 1980s. Tumbak and jurâk are still used to a wide extent but they are not as popular as moassel, particularly among young people and women. Mixing tobacco with molasses is a very ancient habit. A WHO report dates back “the addition of molasses to burley tobacco in the nineteenth century to create “American” blended tobacco”. However, early health-oriented anthropological research on hookah smoking showed that it is much older and can be traced back in the relation by an Arab traveller in India as early as the 17^th^ century [8].

**Narghile:** It is more a Persian/Iranian and Turkish (“narghile” in Turkish)/Middle East word although it has been and is still widely used in the European languages (Italian, French, Romanian, English, *etc*.). Note that in Iran, “narghile” is a water pipe based on a coconut (as a vessel) whereas the present “shisha” would be called “qalyân”.

**Shisha:** It is more an Arabic (Egyptian, Middle East) word although, thanks to the world craze, it is now being used everywhere in the word. It is a word of Persian origin (shishe). It means bottle/recipient (of water). Unlike the “pure” hookah or narghile, the vase of the contemporaneous shisha is made of glass with a typical flask/vial form.

**Tumbak (tumbeki, ‘ajamy):** plain tobacco made of moistened shredded leaves, soaked for hours in water before being squeezed and packed in the bowl of the hookah. As jurak, it is strong (nicotine) and barely used outside Africa and Asia.

**Water pipe:** (in two words or, sometimes, separated by a dash). A general term that is acceptable only when there is one sole form of the object or when artefacts using same smoking preparations are compared. The abuse of this word and particularly its contraction in one word (“waterpipe”) in biomedical research has fuelled a worldwide confusion (scientific nominalism) [7]. For instance, findings of studies carried out in China were inappropriately extrapolated to such different contexts as the Middle East, Europe and the USA [7]. For a comparison, in a publication, researchers would never call “cigarettes” the harm reduction glycerol Eclipse (RJ Reynolds) or the Electrically Heated Accord (Philip Morris) products without giving their specific features (the fact that they heat the tobacco and not burn it; the absence of SSS; *etc.*). Most of the time, this has not been done when “waterpipe” has been used. In a pioneering study on micronuclei in Egypt, experts were led astray because they have used “waterpipe”, not only in the title of their study but also in their questionnaire. However, the reader cannot tell if the subjects had been using goza or shisha which are two different water pipes in Egypt. In the former, the charcoal is directly in contact with the tobacco-molasses based-mixture and therefore heated to a much higher temperature than in the latter (in which the two elements are separated by an aluminium tin foil).

#### Competing interests

The author has never received direct or indirect funding neither from pharmaceutical companies (nicotine “replacement” therapies and products) nor from the tobacco industry. He was the first to publish results of (ambient and alveolar) Carbon Monoxide levels in hookah lounges and their patrons (Tobaccology thesis 1998; Alcoologie 1999; Doctoral thesis 2000) and subsequently issued public health recommendations in this respect. Out of this concern, he has participated in the design of a harm reduction hookah (cutting down CO by 95%) of which he is an official co-inventor (Patent 2005. “Narguilé à allumage simplifié” [Narghile with simplified ignition]. Appl. EP20050291196. Filed 3 June. Published 14 Dec). His participation in the project was frozen by Autumn 2004. However, the official termination was formalised only by 15 June 2005, date by which the author has ceded all his rights regarding the invention (legally certified by State Attorney in Paris). Therefore, the author does not consider this as a competing interest.

## Figures and Tables

**Table 1.** Concentration of particles (millions per mL [median Ø]) through the use of a smoking machine (from Becquemin *et al.* [93]).

Concentration of particles (millions per mL [median Ø])	Cigarette	Narghile
MSS	**3.14** [0.27 μm]	**3.55** [0.34 μm] (before water bubbling) **1.20** [0.27 μm] (after water bubbling)
SSS	**19** [0.09 μm]	**2.91** [0.11 μm]
EMSS (machine)	**2.26**[0.30 μm]	**6.22** [0.25 μm]

**Table 2.** Particles Filter Efficiency (from Bernstein/McCusker *et al.* [61,89]).

Aerodynamic size and filter efficiency of smoke from commercial cigarettes							
Cigarette	Filter type	FTC tar rating^a^(mg/cig)	MMAD (μm) with filter	MMAD (μm) without filter	Number/cm^3^ with filter (10^3^)	Number/cm^3^ without filter (10^3^)	Filter efficiency (%)
IR2F	Cell. acetate	26	0.44	0.43	3.3	4.2	22
Marlboro	Cell. acetate	17	0.43	0.48	3.1	4.5	32
Tareyton	Cell. acetate + charcoal particles	14	0.50	0.47	1.6	4.0	60
Doral II	Cell. acetate + plastic baffles	5	0.50	0.48	1.9	4.4	57
Koolite	Cell. acetate	5	0.43	0.38	1.6	3.9	60
Merit	Cell. acetate	8	0.36	0.38	2.1	3.9	46
Vantage	Cell. acetate	11	0.47	0.48	2.7	5.0	46
Cambridge	Cell. acetate	<1	0.53	0.51	0.25	4.67	96
Barclay	Cell. acetate + vent.holes	<1	0.56	0.36	0.57	5.9	91
Carlton	Cell. acetate + vent.holes	<1	0.43	0.36	0.33	5.3	94
Barclay	Vent.holes taped	–			4.90		
Carlton	Vent.holes taped	–			2.37		

**Abbreviations:** Cell. acetate = cellulose acetate; Vent. Holes = ventilating holes.

**Notes:** From McCusker et al. (1983)

a) Federal Trade Commission, Dec. 1979, Public Health Service, Office on Smoking and Health and National Cancer Institute, DHS Publication No. (PHS) 80-50135; cig, cigarette.

**Table 3.** Estimated dose ratio between active smoking (20 cig./day) and passive smoking (8 h/day) (Scherer et al. [100]).

Tobacco smoke constituents	Smoking (S)(20cig/day)^b^	Passive smoking (PS)(8h/day)^c^	**Dose ratio S/PS**
**GASEOUS PHASE**			
CO (mg)	40–400	14.4–96	2.7–4.2
Formaldehyde (mg)	0.4–1.8	0.08–0.4	4–5
Volatile nitrosamines (μg)	0.05–1.0	0.03–0.4	1.5–2.5
Benzene (μg)	200–1200	40–400	3–5
**PARTICULATE MATTER**			
Particles (mg)	75–300	0.024–0.24	1250–3000
Nicotine (mg) ^d^	7.5–30	0.08–0.4	75–90
Benzo[a]pyrene (μg)	0.15–0.75	0.001–0.011	70–150
Cadmium (μg)	1.5	0.001–0.014	110–1500
Tobacco specific nitrosamines (μg)	4.5–45	0.002–0.010	2300–4500

a) Data are compiled from References 16, 19, 97, 38 (as printed in the original by Scherer *et al.*)

b) Assumed deposition rate for particulate matter: 75% (14)

c) Assumed breathing volume: 0.5 m^3^/h. Assumed deposition rate for particulate matter: 11% (13)

d) Nicotine is particle-bound in MS and a gas phase constituent in ETS (7)

**Table 4.** The 15 Reasons behind the World Upsurge in Hookah (Narghile, Shisha) Smoking. Originally published in the 4^th^ part of the Tetralogy on Hookah and Health: Chaouachi K. *Narghilé: un problema di Sanità Pubblica* [6].

**The 15 Reasons behind the World Upsurge in Hookah (Narghile, Shisha) Smoking**
OBECTIVE REASONS
**1-Global Tourism and Migration Flows** (back from Egypt, Tunisia, **etc.** with a hookah in the suitcase; hookah lounges in the West)
**2- A New Hassle-Free Lighting System** (new easy to light charcoal)
**3-Relative Acceptance by Non-Smokers** (notable smoke irritants filtered out)
**4-Unexepected Backlash Effect of Anti-Tobacco Campaigns** (viewed as safer than cigarette smoking)
**5-Filtration of Some Noxious Substances** (some carcinogens, among others, may be filtered out)
**6-A “Light” Dependence** (seen as easy to quit)
**7-The Influence of Television (case of the Arab World)** (Egyptian movies have featured hookah smokers for decades)
**8-The Rise of Individualism in Modern Societies** (socialising needs and the search for new forms of sociability)
SUBJECTIVE REASONS
**9-Conviviality** (“social” smoking, sharing the hose (ludens), talking, long time passing)
**10-A Powerful Symbolism** (dream, art, “mysticism”, “peace pipe”)
**11-A Transverse Social, Sexual, Religious and Inter-Generational Practice** (social and cultural melting pot)
**12-Flavours** (“tobamel” (mu’assel), a flavoured tobacco (or no-tobacco)-honey/molasses based mixture)
**13-The Cultural Status of Honey** (Koran, *The Bees***)**
**14-A Highly Sensory Experience** (Five senses permanently stimulated)
**15-“Rebellion” Values** (an “anti-modern” concept of time passing in a global world. A social and cultural counter-product of the globalisation process of the Nineties)

http://www.tabaccologia.org/PDF/4_2006/7_42006.pdf

## Abbreviations

CI: Confidence Interval;
EMSS: Exhaled Main-Stream Smoke;
ETS: Environmental Tobacco Smoke (taken as synonym of SHS);
MSS: Main-Stream Smoke;
OTS: (Outdoors Tobacco Smoke);
OR: Odds Ratio;
PM_2.5_: Particle Matter whose size is below 2.5 μm);
PM_10_: Particle Matter whose size is below 10 μm):
PAH: Polycyclic Aromatic Hydrocarbons;
RSP: Respirable Suspended Particles;
SHS: Second Hand Smoke (syn. ETS);
SSS: Side-Stream Smoke;
UFP: Ultra Fine Particles;
WHO: (World Health Organisation).
